# Neolithic dairy farming at the extreme of agriculture in northern Europe

**DOI:** 10.1098/rspb.2014.0819

**Published:** 2014-09-22

**Authors:** Lucy J. E. Cramp, Richard P. Evershed, Mika Lavento, Petri Halinen, Kristiina Mannermaa, Markku Oinonen, Johannes Kettunen, Markus Perola, Päivi Onkamo, Volker Heyd

**Affiliations:** 1 Organic Geochemistry Unit, School of Chemistry, University of Bristol, Cantock's Close, Bristol BS8 1TS, UK; 2 Department of Philosophy, History, Culture and Art Studies, University of Helsinki, PO Box 59, Helsinki 00014, Finland; 3 Finnish Museum of Natural History, University of Helsinki, PO Box 64, Helsinki 00014, Finland; 4 Public Health Genomics Unit, National Institute for Health and Welfare, PO Box 104, Helsinki 00251, Finland; 5 FIMM, The Institute for Molecular Medicine Finland, University of Helsinki, PO Box 20, Helsinki 00014, Finland; 6 Department of Biosciences, University of Helsinki, PO Box 56, Helsinki 00014, Finland; 7 Department of Archaeology and Anthropology, University of Bristol, 43 Woodland Road, Bristol BS8 1UU, UK

**Keywords:** 60th parallel north, dairy farming, biomarker lipids, isotopes, lactase persistence, incoming prehistoric population

## Abstract

The conventional ‘Neolithic package’ comprised animals and plants originally domesticated in the Near East. As farming spread on a generally northwest trajectory across Europe, early pastoralists would have been faced with the challenge of making farming viable in regions in which the organisms were poorly adapted to providing optimal yields or even surviving. Hence, it has long been debated whether Neolithic economies were ever established at the modern limits of agriculture. Here, we examine food residues in pottery, testing a hypothesis that Neolithic farming was practiced beyond the 60th parallel north. Our findings, based on diagnostic biomarker lipids and δ^13^C values of preserved fatty acids, reveal a transition at *ca* 2500 BC from the exploitation of aquatic organisms to processing of ruminant products, specifically milk, confirming farming was practiced at high latitudes. Combining this with genetic, environmental and archaeological information, we demonstrate the origins of dairying probably accompanied an incoming, genetically distinct, population successfully establishing this new subsistence ‘package’.

## 1. Introduction

Since the end of the last Ice Age, some 12 000 years ago, the high northern latitudes of the globe became permanently settled by humans of Late Palaeolithic and/or Mesolithic cultures. Their sole subsistence mode for millennia, and for most of them to the present day, was hunting, fishing and gathering, thereby making use of the plentiful wild resources. While there is no evidence for farming on the North American Continent and in Siberia above the 60th parallel north prior to the European colonization, earlier examples of agro-pastoral farming appear in Iceland in the ninth century AD Viking Age, and an episode (10–15th century AD) in southwest Greenland [1]. In order to make farming viable, these inhabitants of the high northern latitudes had to overcome extreme climatic and environmental conditions. The forced abandonment of the south Greenland settlements at the onset of the Little Ice Age [2] demonstrates the vulnerability of any productive subsistence economy to climate change at these high latitudes. Hence, it has long been doubted whether more ancient prehistoric subsistence economies based on agriculture would have been viable, especially given the limited adaptations in stock animals and domesticated plants, most of which originated in the warm and semi-arid climes of the ‘Fertile Crescent’ of the Levant approximately 11 000 years ago [3]. However, at least in northwestern Europe, thanks to the warming effects of the Gulf Stream, Early Neolithic fourth millennium settlers were reaching as far north as to between the 55th and 58.5th parallel, and probably intermittently beyond, establishing the sustainable farming economies in all of Britain, southern Norway and even east-central Sweden [4–7].

**Figure 1.**
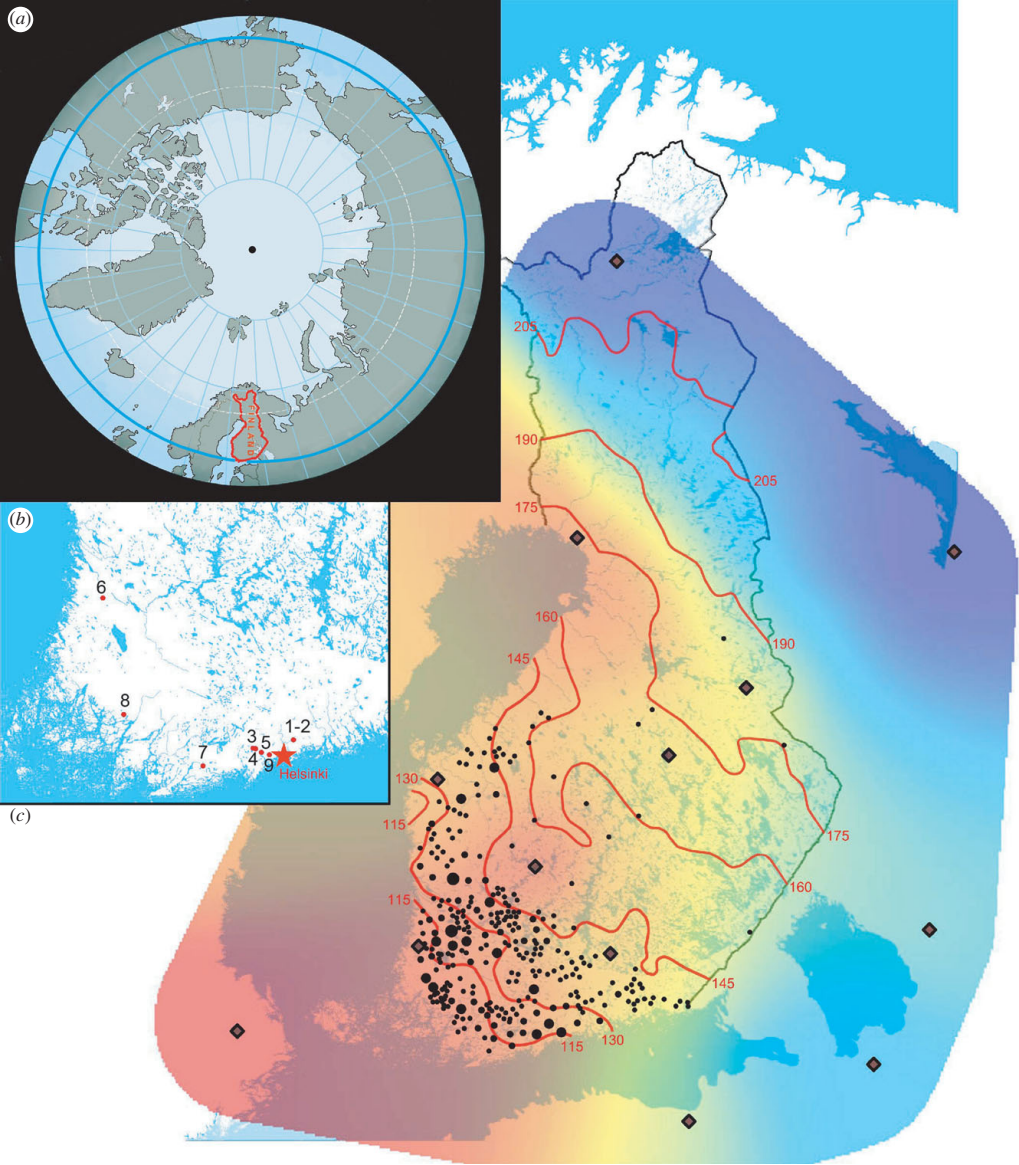
Integrated maps of: (*a*) the northern hemisphere relative to the North Pole. Highlighted are the modern borders of Finland (in red) and the 60th parallel north (in light blue), (*b*) the location of all Finnish prehistoric sites from which sherds were sampled (numbers correspond to table 1), and (*c*) the distribution of the Corded Ware culture within Finland. Mapped (black dots) are finds of typical stone battle axes, used as a proxy (data from [8]). The red isolines indicate average permanent snow cover period from 1981 to 2010 (data from [9]). A recent study estimates the snow cover period *ca* 4500 years ago would have been 40–50 days less than today [10]. Overlying coloration refers to the lactose persistance (LP) allele gradient in modern northeastern Europe (see the electronic supplementary material, appendix B: Material and methods and table 1, for details); lozenge dots specify the dataset mean points for the triangulation.

Here, we explore the possibility for prehistoric farming in Finland at sites located beyond the 60th parallel north. These sites were located at the same high latitude as southern Greenland, Canada's Northwestern Territories, Anchorage in Alaska, Kamchatka Peninsula and near Yakutsk in Siberia, and lying further to the east, were thus exposed to a harsher continental climate. Farming in Finland would have been extremely challenging on account of the low average temperatures and several months of snow cover (figure 1 [8–10]) limiting vegetation periods [11]. The year was often interrupted by cold spells with snow and ice even in summertime, such that cereal agriculture is nowadays only just possible, and stock require considerable periods of shelter and foddering during the long winters. The date of the earliest practices of domestication at this latitude in Europe has been questionable owing to the paucity of surviving cultural and biological evidence from the prehistoric period. There is, at present, neither evidence to suggest that animal domestication was established during the climatic and demographic optimum of the first half of the fourth millennium BC [12], nor even that it was associated with the subsequent appearance of people using pottery belonging to the Pan-European Corded Ware phenomenon in the third millennium BC. Indeed, it appears to have a much later date [13], despite the people associated with the latter culture being strongly associated with pastoral farming economies elsewhere in Europe [14], and who were thought to have carried with them the ability to digest milk into adulthood (lactase persistence, LP) into southern Finland in the third millennium BC [15]. Nowadays, both the prevalence of LP and consumption of dairy products in this part of northern Europe are among the highest in the world [16,17].

The exceptionally poor survival of archaeological remains in the acidic soils of southern Finland normally only leave small pieces of burnt (cremated) animal bones for further analysis [18], and with macrofossil plant remains never systematically investigated, it has thus far been impossible to reconstruct whether these pioneer Corded Ware ‘pastoral farmers' were ever able to establish farming above the 60th parallel north or whether there was a return to the plentiful wild resources, driven by the harsh climatic conditions [19,20]. To date, the earliest domesticate bone recovered from southern Finland is a sheep/goat dated to the Final ‘Neolithic’ Kiukainen culture, *ca* 2200–1950 BC [18], with the earliest cattle and horse not dated earlier than the Bronze Age [18]. Infrequently recovered domesticate bones from potential Corded Ware contexts have recently been directly dated to the historic or modern period [18].

Fortunately, the acidic soils that preclude survival of bones have the advantage of offering favourable conditions for the survival of certain classes of ancient biomolecules, such as lipids in the walls of ancient ceramic cooking vessels, represented by sherds recovered in considerable numbers. The carbon isotopic compositions of such biomolecules can be used to assign organic residues to their origins, in particular, to distinguish aquatic fats from those of terrestrial species, and dairy fats from carcass fats [21,22]. Additionally, specific diagnostic biomarkers that survive include isoprenoid fatty acids originating from marine organisms and long-chain ω-(*o*-alkylphenyl)alkanoic acids (APAAs) and vicinal diols (DHYAs) that arise from heating or oxidation of the highly reactive mono- and polyunsaturated fatty acids, characteristically found in abundance in aquatic fats [23–25]. Based on the above biomarkers and carbon isotope proxies, we now have tools to allow us to robustly investigate the economy and pottery function of prehistoric hunter–fisher–foragers (people using so-called Comb Ware) and the potentially earliest farmers (so-called Corded Ware, Final ‘Neolithic’ Kiukainen Ware and Early Metal Age prehistoric pottery people) and explore their inter-relationship with the environment.

Settlement sites from which we obtained pottery sherds for biomarker lipids and isotopes analyses are located in southern and southwestern Finland, all being north of the 60th parallel (see the electronic supplementary material, appendix A, for details and figure 1*b* for their exact geographical location). We have chosen these sites owing to their importance in Finnish prehistoric research, their excavated archaeological features, relative abundance and good preservation of pottery remains, and chronological range spanning from the fourth to the first millennium BC. These are the Typical/Late Comb Ware (fourth millennium BC) site of Vantaa Stenkulla/Maarinkunnas; the Corded Ware (third millennium BC) sites of Tengå Nyåker, Koivistosveden and Backisåker 1 (Kvarnåker), all near the southern Finnish town of Kirkkonummi; the Kiukainen Ware (around 2000 BC) site of Nakkila Uotinmäki, near the town of Pori in southwest Finland; the Late Bronze Age (around 1000 BC) sites of Raasepori Kroggård Hagnäs llb and Kaarina Toivola Hulkkio in southwestern Finland; and the Morby Ware (first millennium BC) site of Espoo Bolarskog I. As is typical for this region, few if any, identifiable fragments of animal bone were reported (electronic supplementary material, appendix A).

## 2. Results

**Figure 2.**
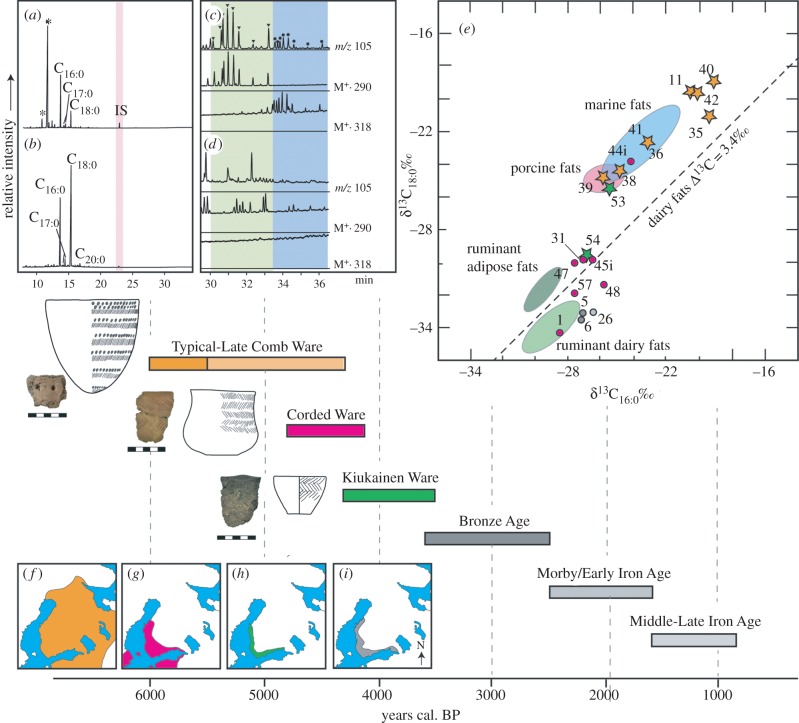
Lipid compositions, aquatic biomarker distributions and stable isotope values of extracts from prehistoric sherds. Typical partial gas chromatograms of lipid extracts from (*a*) Comb Ware and (*b*) Corded Ware; CX:Y FA denotes fatty acid with carbon chain length X and degree of unsaturation Y, *denotes phthalate. Panels (*c*) and (*d*) are mass chromatograms from Comb and Corded Ware lipid extracts, respectively, analysed by GC/MS-SIM, showing the distribution of C_18_ (inverted triangle) and C_20_ (black circle) APAAs present only in (*c*). Panel (*e*) shows δ^13^C_16:0_ and δ^13^C_18:0_ values from Typical/Late Comb Ware (orange), Corded Ware (pink), Kiukainen Ware (green) and Metal Age (grey) residues; when shown as stars, this indicates aquatic biomarkers were also observed in the residue. Numbers refer to the KM-number, as assigned in table 1. Shaded reference ellipses derive from modern reference fats [21,22]. The timeline shows the archaeological cultures discussed here alongside actual sherds sampled and typical vessel forms (after [26–28]) (latter not shown to scale). Distribution maps show the geographical range of (*f*) Typical Comb Ware, (*g*) Corded Ware, (*h*) Kiukainen Ware and (*i*) Bronze Age cultures in the region (after [10,20,29]).

Seventy prehistoric sherds were investigated according to well-established analytical procedures described in the Material and methods. Well-preserved lipids were recovered from 19 sherds. These include Comb Ware sherds deriving from the multiphase site of Vantaa Stenkulla/Maarinkunnas (table 1), dating to *ca* 3900–3300 cal. BC, at which time the settlement was located at a narrow Litorina Sea bay opening to a second outer bay. Subsistence was probably based upon a hunting–fishing–foraging subsistence economy, with the recovered faunal remains and fishing equipment suggesting a significant role of marine resources. The lipid residues from the Comb Ware pointed- and round-base pots all originate from a predominantly or exclusively marine origin, displaying high concentrations of palmitic acid (figure 2 [26–29]), enriched carbon isotope signatures, long-chain APAAs and DHYAs and isoprenoid acids. The lipid residues thus suggest highly specialized subsistence strategies and/or specialized or selective vessel use for processing marine commodities, possibly for storage or exchange [30]. Although it has been debated whether Typical Comb Ware pottery would have been able to withstand cooking, the formation of APAAs requires temperatures of approximately 270°C [21,23,25] and therefore processing of marine products using heat seems highly likely. Comb Ware settlements, faunal assemblages and the size and fragility of Comb Ware vessels suggest that these populations were probably sedentary. A specialized economy based upon coastal resources in close proximity would have permitted such reduced mobility, while the use of pots would have facilitated heat-processing and storage from episodes of over-killing. It is therefore likely that there was a very close inter-reliance between subsistence patterns, frequent pottery use and sedentism.

**Table 1.** Description of sherds containing significant concentrations of preserved lipids. (Site descriptions are given in the electronic supplementary material, appendix A. H.E.S. bowl, half egg-shaped bowl; FFAs, free fatty acids; APAAs, ω-(*o*-alkylphenyl)alkanoic acids; DHYAs, dihydroxy acids; TMTD, 4,8,12-trimethyltridecanoic acid; Phy, phytanic acid; Pris, pristanic acid.)

map no.	lab. code	lipid conc (μg g^−1^)	form	δ^3^C_16:0_	δ^3^C_16:0_	Δ^13^C	lipid composition	classification
Typical/Late Comb Ware								
1	*Vantaa Maarinkunnas*							
	KM-11, 30464 : 380	209	bowl	−20.4	−19.5	0.9	FFAs (C_14_–C_20_); C_18_–C_22_ APAAs; C_18_–C_22_ DHYAs	marine fat
2	*Vantaa Stenkulla*							
	KM-35 29954 : 2128	565	H.E.S bowl	−19.4	−21.1	−1.7	FFAs (C_14_–C_22_); C_18_–C_22_ APAAs; TMTD, Phy; C_18_–C_20_ DHYAs	marine fat
	KM-36 29954 : 2357	151	H.E.S bowl	−23.2	−23.1	0.1	FFAs (C_14_–C_20_); C_18_–C_20_ APAAs; Phy; C_18_–C_20_ DHYAs	marine fat
	KM-38 29954 : 3105	60	H.E.S bowl	−24.8	−24.4	0.4	FFAs (C_12_–C_20_); C_18_–C_20_ APAAs; TMTD, Pris, Phy; C_18_–C_20_ DHYAs	marine fat
	KM-39 29954 : 4932	273	H.E.S bowl	−25.8	−24.8	1.0	FFAs (C_12_–C_20_); C_18_–C_22_ APAAs; TMTD, Pris, Phy; C_18_–C_20_ DHYAs	marine fat
	KM-40 29954 : 7738	146	H.E.S bowl	−19.1	−19.0	0.2	FFAs (C_14_–C_20_); C_18_–C_20_ APAAs; TMTD, Phy; C_18_–C_20_ DHYAs	marine fat
	KM-41 29954 : 9074	204	H.E.S bowl	−23.2	−22.7	0.5	FFAs (C_14_–C_20_); C_18_–C_20_ APAAs; TMTD, Pris, Phy; C_18_–C_20_ DHYAs	marine fat
	KM-42 29954 : 1840	66	H.E.S bowl	−20.2	−19.6	0.5	FFAs (C_14_–C_20_); C_18_–C_20_ APAAs; TMTD, ?Pris, Phy	marine fat
Corded Ware								
3	*Kirkkonummi Tengå Nyåker*							
	KM-1 8709 : 52	2398	beaker	−28.6	−34.3	−5.8	FFAs (C_14_–C_24_)	dairy fat
	KM-31 8709 : 35	342	‘S-shaped’ amphora	−27.1	−29.8	−2.7	FFAs (C_12_–C_20_); TMTD, ?Phy	ruminant carcass fat
	KM-47 8709 : 17	105	large beaker, impressed decoration	−27.7	−30.0	−2.3	FFAs (C_14_–C_18_)	ruminant carcass fat
	KM-48 8709 : 22	292	large beaker	−25.8	−31.4	−5.5	FFAs (C_12_–C_22_); C_18_ DHYAs	dairy fat
4	*Kirkkonummi Koivistosveden*							
	KM-44i 7734 : 11	1305	beaker	−24.2	−23.8	0.4	FFAs (C_14_–C_20_); C_18_–C_20_ DHYAs	?marine fat
5	*Kirkkonummi Backisåker I (Kvarnåker)*							
	KM-45i 7349 : 5	57	large ‘S-shaped’ amphora	−26.5	−29.8	−3.3	FFAs (C_14_–C_22_); C_18_ DHYAs	ruminant carcass fat
	KM-57 5944 : 46	1826	decorated beaker	−27.7	−31.8	−4.1	FFAs (C_12_–C_24_), ?Phy	dairy fat
Kiukainen Ware								
6	*Nakkila (Kiukainen) Uotinmäki*							
	KM-53 5942 : 9	484	large funnel-type	−25.4	−25.6	−0.1	FFAs (C_14_–C_18_); C_18_–C_20_ APAAs; TMTD, ?Pris, Phy; C_18_–C_20_ DHYAs	marine and ruminant fat
	KM-54 5942 : 11	323	unknown	−26.9	−29.7	−2.8	FFAs (C_12_–C_20_); C_18_–?C_20_ APAAs;?TMTD, Phy; ?C_18_ DHYAs	ruminant fat, ?marine fat
Late Bronze Age								
7	*Raasepori (Karjaa) Kroggård Hagnäs llb*							
	KM-5 20872 : 161	5851	amphora-type, flat bottom	−27.2	−33.2	−6.0	FFAs (C_14_–C_24_)	dairy fat
8	*Kaarina Toivola Hulkkio*							
	KM-6 27793 : 89	233	bowl with flat bottom	−27.3	−33.6	−6.3	FFAs (C_12_–C_20_)	dairy fat
Morby Ware (Late Bronze Age/Early Iron Age)								
9	*Espoo Bolarskog I*							
	KM-26 15583 : 7	1372	small roundish pot with flat bottom	−26.5	−33.1	−6.6	FFAs (C_14_–C_24_)	dairy fat

Three sites of the Corded Ware culture yielded preserved organic residues. No faunal remains were reported from any of these sites, with the exception of a single fragment of burnt wild mammal bone from *Tengå Nyåker* [27]. However, in contrast with the Comb Ware sherds, the organic residues preserved in diagnostic Corded Ware sherds from sites at Kirkkonummi (*Tengå Nyåker* and *Backisåker*), dated to *ca* 2500 cal. BC, display stable carbon isotope signatures typical of the fats of terrestrial ruminants, despite their locations being less than 2 km from the contemporary coastline [31]. While theoretically, the stable carbon isotope values could originate from domesticated (e.g. cattle) or wild (e.g. elk, forest reindeer) ruminants, half of these residues are milk fats, which must have originated from domesticated stock (figure 2). Intriguingly, the three dairy fat residues were all associated with beaker-type ‘drinking’ vessels, often occurring in grave deposits, and not with the amphorae and S-shaped pots more typical of settlements.

Only one residue from a Corded Ware vessel, deriving from a third site at Kirkkonummi (*Koivistosveden*), contained fatty acids exhibiting enriched stable carbon isotope values and long-chain DHYAs, indicating a marine origin. Although lying less than 2 km from the aforementioned Corded Ware sites, *Koivistosveden* would have had the closest proximity to the contemporary coastline and demonstrates that pottery vessels were not used exclusively for terrestrial resources by Corded Ware users.

The Final ‘Neolithic’ Kiukainen culture, whose ceramic inventory shows similarities with Late Corded Ware and local hunter–fisher–forager ware (Pyheensilta Late Comb Ware), is believed to be a cultural amalgamation emerging locally during a period of climatic deterioration [20,32]. While the low number of residues recovered makes interpretation preliminary, this intriguingly appears mirrored in the pottery residues, because the fatty acid stable carbon isotope values fall along a mixing line between ruminant and non-ruminant/marine products. Although the isotope signatures reflect at least some contribution of terrestrial fat, both residues contain biomarkers for aquatic fats, including long-chain APAAs and isoprenoid fatty acids. As neither the Comb Ware, nor the Corded Ware pottery residues exhibited evidence for mixing of terrestrial and aquatic products, these findings indicate either that this culture practiced a less-specialized economy, perhaps re-introducing aquatic resources as a buffer against deteriorating or fluctuating climatic conditions, or that use of vessels for varied purposes was now practiced.

Finally, residues from Early Metal Age pottery (*ca* 1200–500 BC) all derived from dairy fats. Increasing population size despite the continuing climatic deterioration of the Late Holocene is believed to have arisen from the intensification of agriculture by the later Metal Ages [33] which overcame environmental constraints upon population size. Certainly, such a scenario of established stock-rearing would be supported by the prevalence of dairy fats in the pots.

## 3. Discussion

It has been observed that the global prevalence of the LP phenotype is associated with cultures with a history of milk exploitation, with patchy distributions of LP in Africa and the Middle East associated with nomadic pastoralists, in contrast to their non-pastoralist neighbours [34]. These findings presented here demonstrate the antiquity of dairy product processing in southern and southwestern Finland, a tradition reflected by both the high frequency [35] and distribution of the LP allele in present-day Finland. The SW–NE gradient in the frequency of the LP allele in Finland (figure 1) is the product of recurrent, substantial immigrations from the west and east over the past 6000 years [15,36] and its highest frequency exhibits close correlation with the distribution of Corded Ware settlements in southern and southwestern Finland. Genetic evidence suggests that low frequencies of LP in some parts of the eastern Baltic may reflect long-lasting ‘genetic refugia’ for hunter–fisher–forager populations [37]. However, the age estimate for the only LP haplotype in Finland, H98 containing the T-13910 allele shows divergence *ca* 5000 years ago [38]. This is consistent with a correlation between immigrating Corded Ware people, their milk use in the far north and the probable first appearance of the LP, still reflected in the LP gradient of modern-day Finland more than 4500 years later.

Our investigations into organic residues preserved in hunter–fisher–forager and ‘early farmer’ pottery vessels from Finland provide, to our knowledge, the first direct evidence that animal domestication, specifically including dairy production, was practiced by early prehistoric farmers beyond the 60th parallel north. With the earliest directly dated domesticate bone currently dating to the Kiukainen culture, at least 500 years later [18], the identification of dairy fats associated with Corded Ware pottery now pushes the date for domestication back to *ca* 2500 BC and for the first time, directly associates the appearance of a new cultural horizon with the arrival of animal domestication. The strong contrast between the marine products processed in hunter–fisher–forager Comb Wares and terrestrial and domesticated secondary products processed in Corded Wares supports the hypothesis that Corded Ware pottery represents the successful introduction of novel subsistence practices into Finland and, moreover, places the prehistoric origins of farming and milk consumption, at the most northerly latitudes so far, some 4500 years ago. However, the biomarker and stable isotope compositions of residues from the Final ‘Neolithic’ Kiukainen period tentatively indicate reversion to aquatic foods probably associated with episodic climate deterioration [10,39] showing vividly the vulnerability of any early farming system.

When viewed alongside evidence for dairying from other parts of northern and northwestern Europe [5,7,22,40], the importance of milk, cattle and stock-keeping, alongside cereal agriculture, in the demic farming colonization of Europe's northern latitudes [41] is unequivocally established. But whereas in northern Britain, a terrestrial subsistence economy remains the sole food source for more than 1500 years after the initial colonization [5], southern Scandinavia shows the continuation of the exploitation of aquatic resources as an additional food resource alongside agricultural products [7,40]. A third more opportunistic way may have been chosen by the Late Corded Ware inhabitants of Finland, and their Kiukainen successors, by adopting again hunting–fishing–gathering practices after some generations as the later third millennium BC annual temperatures continued to fall [42].

This rather episodic character of prehistoric farming is probably symptomatic of cultivation in marginal landscapes above the 60th parallel north also evident later in Bronze and Iron Age records [13,33,43] and in historical times [44]. Even today, Finland is one of the most northerly agricultural zones of Europe and the inhabitants of northerly latitudes have to overcome unfavourable extreme climatic conditions to make ‘conventional’ farming viable [11]. Although predicted global warming raises the possibility of modern-day populations extending agriculture to higher latitudes in the future on a global scale, our results show how climatic instabilities at such frontier zones will make continuous farming extremely challenging [45,46].

## 4. Material and methods

The protocol briefly comprised cleaning of a small portion of the external surfaces of the potsherd using a modelling drill and the removal of this cleaned piece using a chisel. Cleaned sherd fragments were crushed in a solvent-washed mortar and pestle and an internal standard added (20 μg *n*-tetratriacontane) prior to solvent-extraction using 2 × 10 ml CHCl_3_/MeOH 2 : 1 v/v via sonication (20 min). After centrifugation, the solvent was decanted and blown down to dryness under a gentle stream of N_2_. Aliquots of the total lipid extract were filtered through a silica column and treated with 40 µl N,O-*bis*(trimethylsilyl)trifluoroacetamide (BSTFA, 70°, 1 h) prior to screening using high-temperature gas chromatography (GC).

Aliquots from selected sherds were then hydrolysed (0.5 M NaOH/MeOH; 70°, 1 h) and methylated (100 µl BF_3_/MeOH; 75°, 1 h) for the structural identification of components using GC/mass spectrometry (GC/MS) and highly sensitive detection of specific biomarkers using selected ion monitoring (GC/MS-SIM; scanning for ions *m*/*z* 105, 262, 290, 318 and 346). The isotopic composition of individual fatty acids was determined using GC-combustion-isotope ratio MS (GC/C/IRMS). The δ^13^C values were derived according to the following expression and are relative to the international standard vPDB: δ^13^C ‰ = ((*R* sample – *R* standard)/*R* standard) × 1000, where *R* = ^13^C/^12^C. The δ^13^C values were corrected for the carbon atoms added during methylation using a mass balance equation [47].

The ‘bound’ fraction from selected sherds was released through the alkaline extraction of solvent-extracted pottery, using 5 ml 0.5 M NaOH/MeOH in DCM-extracted double-distilled water (9 : 1 v/v; 70°, 1 h). After acidification to pH3, these ‘bound’ lipids were extracted using 3 × 3 ml DCM. The bound fraction was treated with 40 μl BSTFA (70°, 1 h) prior to analysis using a GC/MS fitted with a non-polar column, operated in full scan and SIM mode (*m*/*z* 215, 317, 517, 345, 545, 243 and 573).

## Supplementary Material

Appendices

## Data accessibility

Archaeological GC and GC/MS data: Bristol University Research Data Repository (doi:10.5523/bris.13kidnrls4jnl1m806eyfd8h6z). Archaeological single compound stable isotope data: Bristol University Research Data Repository (doi:10.5523/bris.upjtf9os1dzr154phmgvrupib).
