# Supplementary material for: Neolithic dairy farming at the extreme of agriculture in northern Europe
Source: Proc Biol Sci. 2014 Sep 22;281(1791):20140819. doi: 10.1098/rspb.2014.0819 (PMC4132672; doi:10.1098/rspb.2014.0819)
Supplement: Appendices [file rspb20140819supp1.pdf]

Appendices

Appendix A. A brief description of the sampled sites. The numbers refer to geographical location shown in Figure 1.

Typical / Late Comb Ware

1-2) Vantaa Stenkulla/Maarinkunnas[48]

Coordinates: P (YKJ): 6688736 I (YKJ): 3393380, P (YKJ): 6688640 I (YKJ): 3393238; 20-35 m.a.s.l.

Several excavations were conducted by various archaeologists during 1979-1996 at the site of Vantaa *Stenkulla-Maarinkunnas* (Southern Finland). It was an extensive coastal site, situated at a narrow Litorina Sea bay. The location was favourable, close to the shallow archipelago and the open sea, and it also had waterways to the North by the rivers. The Stone Age settlement consists of remains of multiple dwellings, hearths, and one red-ochre grave. Ceramics are mainly Typical and Late Comb Ware, but pieces of Corded Ware and Asbestos Ware have also been found. Artefacts are variable: ceramics, flint tools, flint arrow heads, fishing hooks, line weights, hazel nut shells, amber artefacts, clay-idols, etc. Burnt bone material (total 1536 identified fragments) revealed a variety of wild animals, mainly seals (Phocidae), Eurasian Elk (*Alces alces*) and Eurasian beaver (*Castor fiber*). AMS-dates from Stenkulla are 5020±110 BP (charcoal), 5000±120 BP (charcoal), 4970±90 cal BP (charcoal), 4980±100 BP (charcoal), 910±90 BP (charcoal). One red ochre grave probably dates to the Typical Comb Ware. Finds are catalogued within the National Museum Numbers (NM) 30464 and 29954.

Corded Ware sites

3) Kirkkonummi Tengå Nyåker[27]

Coordinates: P (YKJ): 6681465 I (YKJ): 3358830; 35 m.a.s.l.

The site is situated on a gentle slope opening towards the southeast and Lake Lapinkylänjärvi which was isolated from the Baltic Sea at the time of the occupation. The distance from the site to the lake is about 300 m and 2100 m to the former shore of the Baltic Sea. A river flows from Lake Lapinkylänjärvi to Lake Loojärvi. It may have formed a narrow bay of the Baltic Sea during the occupation of the site. Finds comprise pottery sherds and some stone implements, such as axes, adzes and quartz fragments and a single fragment of burnt wild mammal bone. However ceramics are by far in the majority and it is important to note that all so far only belong to the Corded Ware culture. Aarne Europaeus (Äyräpää), the most important scholar on Corded Ware in Finland, excavated the site in 1926. Torsten Edgren then carried out a second excavation in 1982. Thus far, the most recent excavation was led by Kreetta Lessell in 2011. Finds are catalogued within the National Museum Number (NM) 8709.

4) Kirkkonummi Koivistosveden[49]

Coordinates: P (YKJ): 6681086 I (YKJ): 3360996; 35 m.a.s.l.

The site is situated on a slope towards the northeast and Lake Loojärvi, which formed a narrow bay of the Baltic Sea, close to the end of the bay. The distance from the site to the shore of the sea was ca. 100 m. Behind the site is a high hill protecting the settlement from

the southerly sea winds. The finds from the site are rich and versatile, including quartzes, stone implements, such as axes, adzes and whetstones, and ceramics. No identified faunal bones were reported. Ceramic types mostly belong to the Corded Ware, but some textile-impressed pottery was also found. The dwelling site of Koivistosveden was never excavated; all artefacts were collected during field surveys carried out by Aarne Europaeus in 1920 and Veikko Lehtosalo in 1963. ^{14}C-dating was therefore never applied here. Finds are catalogued within the National Museum Numbers (NM) 7734 and 9107.

5) Kirkkonummi Backisåker 1 (Kvarnåker)[27]

Coordinates: P (YKJ): 6678014 I (YKJ): 3365883; 35 m.a.s.l.

The site is situated on the northern side of the river running from Lake Lapinkylänjärvi to Lake Loojärvi. The distance to both lakes is about 600 m today. During the Corded Ware period the river flowed to the narrow bay of the Baltic Sea. Behind the site, on its northern side, there is a *ca*. 20 m high hill, formed of bedrock. The dwelling site is mostly represented by the material culture of the Corded Ware period. The find material is extensive and includes, besides pottery, stone axes, adzes and quartz implements. No faunal remains were reported. An initial excavation at the site was carried out by Guy Topelius already at the beginning of the 1900s. Aarne Europaeus was aware of this important site but had no possibility to excavate further. Later archaeological fieldwork was restricted to surveys and occasional inspections. Nowadays it seems most of the site is destroyed as the result of construction and agricultural activities in later 20th century. There are no ^{14}C dates. Finds are catalogued within the National Museum Numbers (NM) 5944, 6139 and 7349.

Kiukainen Ware

6) Nakkila (Kiukainen) Uotinmäki[50]

Coordinates: P (YKJ): 6809524 I (YKJ): 3230653; 23-37,5 m.a.s.l.

The site is known in Finland as a settlement of the Late 'Neolithic' but also as a Bronze Age cairn monument. The landscape belongs to an esker of Hiittenharju running in SE-NE – direction following the contemporary Kokemäenjoki river. At the time of the Kiukainen culture at the very end of the 'Neolithic' it formed a bay of the Baltic Sea. The site includes the dwelling site itself and a total of nine cairns. Finds from the Bronze Age cairns only consisted of burnt bones including cattle and sheep/goat and one bronze object. The material culture of the dwelling site is mostly represented by ceramics of the Kiukainen type, however also a few Late Bronze Age or, more likely, Pre-Roman Iron Age sherds are present. No Corded Ware was found on the site. In addition to the pottery, stone axes, adzes of different types, rhomb-formed axes and cubic-formed stones are also retrieved. The first excavations of the cairns were carried out in 1897 by H.J. Heikel and further fieldwork followed by Julius Ailio in 1903. Since then, only surveys and occasional inspections were made in the area. The finds are catalogued within the National Museum Numbers (NM) 5942 and 5942.

Late Bronze Age

7) Raasepori (Karjaa) Kroggård Hagnäs llb[51]

Coordinates: P (YKJ): 6666383 I (YKJ): 3316109; 22-25 m.a.s.l.

Kroggårds Hagnäs llb is a dwelling site today lying mainly on the NE side of a gently descending cornfield at the crossroads of the Löwenstreet and the Hanko-Hyvinkää-road no.

53. However, considerable parts of the site are now destroyed due to roadworks and cultivation. The site is naturally protected by a slope on its NW and SE sides. Nowadays further away, its distance to the Baltic Sea may have been only *ca*. 100 m in the Corded Ware period. The occupation history of the site is complex according to the findings: earliest ceramics belong to Late Comb Ware, followed by Corded Ware and then textile-impressed pottery and some sort of Epi-Neolithic ceramics. From the Late Iron Age the whole area appears only thinly populated. At the site, crop rotation was applied. Rescue excavations were carried out in 1982: Tuula Heikkurinen and Esa Suominen excavated the immediate crossroad and all parts to be destroyed by the road. The rest of the site was only surveyed. Finds are catalogued within the National Museum Numbers (NM) 20872.

8) Kaarina Toivola Hulkkio[52-53]

Coordinates: P (YKJ): 6710275 I (YKJ): 3248483; 24.5-25 m.a.s.l.

Kaarina Toivola Hulkkio, located in Southern Finland, is a dwelling site with several potential long house remains and related artefacts (59 postholes, hearths, pottery, flint, quartz, burnt clay daub and stone artefacts). In the Bronze Age the site was located in a bottom of a shallow Litorina Sea bay, protected by small islets. The site was excavated by Nina Strandberg in 1992-1993. The dating falls mainly in the Early Metal Age period; Morby and Lusatian Wares, *ca.* 2400 ± 55 BP. The sparse faunal remains include a few burnt fragments of possible cattle, sheep/goat and dog, although these are questionable[18]. Palynological analysis revealed barley (*Hordeum vulgare*) and lymegrass (*Leymus arenarius*). Finds are catalogued within the National Museum Numbers (NM) 27175 and 27793.

Morby Ware (Late Bronze Age/Early Iron Age)

9) Espoo Bolarskog I[54]

Coordinates: P (YKJ): 6676007 I (YKJ): 3372701; 14-16 m.a.s.l.

Excavations were conducted in 1964 by Carl-Fredrik Meinander and 1972-1974 by Arja Nissinen. The site consists of finds that can be connected to the dwelling (e.g., sherds of Morby and Dåvits types, quartz, burnt bone) and a burial cairn. The site dates to the Early Metal Age period; a radiocarbon date from charcoal from a hearth gave a date 510 BC and a cairn 840 BC. Finds are catalogued within the National Museum Numbers (NM) 15583.

Appendix B. Materials and methods for Lactase persistence (LP) frequency estimates

In North European populations, LP is due to polymorphism C/T_{-13910} which is located approx. 14 kb upstream from the start codon of the lactase gene. The persistent individuals have genotype TT or CT.

The SNP LCT C/T-13910 was genotyped using the iPlex assay on the MassARRAY System (Sequenom, San Diego, CA, USA) using standard protocols in Health2000; a general health examination and interview survey collected in year 2000[55]. Health2000 included 5320 individuals collected across Finland. The region of residence information was used to calculate the regional allele frequency estimates for rs4988235 in Figure 1 for Oulu, Kuopio, Tampere, Turku and Helsinki and Southeast Finland. Other frequency estimates in Figure 1 are from published data[56-61]. Numbers of individuals and allele frequencies used for the lozenge dots in Figure 1 are listed in Appendix Table.

Appendix Table. Lactase Persistence T-allele frequency in various regions of Eastern Fennoscandia, Estonia and parts of modern Northwest Russia.

**Region**	**T-allele Frequency**	**N of Individuals**	**Source**
Helsinki and Southeast	0.60	1944	*This study*
Turku	0.68	857	*This study*
Tampere	0.68	1427	*This study*
Kuopio	0.58	1089	*This study*
Oulu	0.66	881	*This study*
Saami	0.17 (±0.04)	30	[38]
Estonia	0.50	355	[59]
Sweden	0,72	1673	[58]
Udmurt	0,26	94	[60]
Komi-Permyaks	0,36	69	[60]
Northwest Russia, Archangelsk region	0,35	34	[61]
North Karelia	0.56	571	[38,57]
